# Validation of the family health scale among heterosexual couples: a dyadic analysis

**DOI:** 10.1186/s12889-022-12499-0

**Published:** 2022-01-13

**Authors:** AliceAnn Crandall, Melissa Barlow

**Affiliations:** Department of Public Health, Brigham Young University, 4103 Life Sciences Building, Provo, UT 846602 USA

**Keywords:** Families, Family health, Psychometrics, Scale validation, Dyadic analysis

## Abstract

**Background:**

The Family Health Scale (FHS) is a recently validated comprehensive measure of family health for use in survey research with the potential to also be used as a clinical measure. However, previous research has only validated the FHS among one member of the family rather than multiple family members. The objective of the study was to examine the psychometric properties of the FHS long- and short-form among married and cohabitating partners (dyads).

**Method:**

The sample for this study was comprised of 482 married or cohabitating heterosexual couples (dyads) who were parents of a child between the ages of 3–13, heterosexual, and living in the United States. Each member of the dyad completed a survey about his or her perception of family health, personal health, childhood experiences, and demographic characteristics. Confirmatory factor analyses (CFA) were conducted to examine the factor structure. Unidimensional, correlational, and second-order factor structures were examined using responses from both partners. The relationships between family health with individual health and demographic covariates were also examined.

**Results:**

Women and men reported their family health similarly. The unidimensional factor structure had the best fit for the FHS short-form while either the unidimensional model or the second-order model would be appropriate for the FHS long-form. Household income, individual member mental health, and childhood experiences were associated with family health in the expected direction.

**Conclusion:**

The results demonstrate that the FHS is a valid and reliable family measure when examining family health among dyads including married and cohabitating heterosexual couples who have children.

## Introduction

Family health is “a *resource* at the level of the family unit that develops from the intersection of the health of each family member, their interactions and capacities, as well as the family’s physical, social, emotional, economic, and medical resources” [1]. Strong family health allows families to fulfill their functions and develop resilience in the face of challenges whereas less healthy families struggle to perform some or many of their functions and may struggle more in the face of adversity. Thus, healthy families promote individual health and development throughout the course of life [2–4]. In fact, the family may be the most salient system for predicting individual health [4]. For decades many have recognized the need to examine the health of the family in healthcare delivery and other settings in an effort to provide better care for individual members [5]. However, one of the challenges with examining family health was that until recently there was no validated comprehensive measure of family health [1, 6].

A variety of validated survey instruments measure aspects of family wellbeing, such as family cohesion, family functioning, or family resilience. These measures have sometimes been conflated with family health. However, while undoubtedly these other family measures may have similarities with family health, they are either aspects of a dimension of family health (e.g., good family communication) or outcomes of family health (e.g., family functioning and resilience). Understanding family health requires one to look at the family from the viewpoint of multiple disciplines [1, 7, 8]. Through an interdisciplinary lens, one can consider a variety of family-level factors that comprise family health such as communication and problem-solving skills, routines, mental and physical health of individual members, familial behavioral habits, social and emotional support available to the family, material resources, family help-seeking efficacy, health insurance, access to external resources, among other things [1, 6, 8].

The Family Health Scale (FHS) is a recently validated comprehensive measure of family health for use in survey research with the potential to also be used as a clinical measure [6]. The FHS includes 32 items covering four domains: family social and emotional health processes, family healthy lifestyle, family health resources, and family external supports. A 10-item short-form version of the FHS incorporates all four domains into a unidimensional scale. The initial scale validation of the FHS was conducted among adults in the U.S. coming from a variety of family types and was found to be both reliable and valid [6]. Extant literature using this scale is limited given the newness of the measure, but childhood family experiences appear to be predictive of family health in adulthood across all four domains [9], and adult depressive symptoms are strongly negatively associated with family health across each domain of the long-form [10] and the short-form [6]. However, an important limitation of this prior research using the FHS is that the scale was only validated among one member of the family. To be a true family measure, research is needed that involves multiple members of the same family all reporting on their family health.

To address this gap, the aim of the current study was to examine the psychometric properties of the FHS long-form (FHS-LF) and short-form (FHS-SF) among married and cohabitating heterosexual partners (dyads) who were parents and living in the United States. Given that prior research has shown that the items are largely measurement invariant across genders [6], we hypothesized that both members of the dyad would report their family health similarly. A secondary aim was to explore how men’s and women’s characteristics (e.g., demographics, childhood experiences, adult mental health) were associated with their family health. We hypothesized that individual member health would be strongly correlated with family health and that demographic characteristics would have only modest effects on family health.

## Methods

The sample was recruited from the Family Health Explorations study. The Family Health Explorations was comprised of 508 married or cohabitating dyads who were recruited into the study using a Qualtrics panel. Inclusion criteria included that the dyads had to be parents of a child between 3 and 13 years of age, live in the United States, and both partners had to be willing to take the survey. A subset of the panel was required to be from a racial minority group (at least one member of the dyad was non-white) and another subset was low education (at least one member of the dyad with less than a high school education). Because the sample size of same-sex couples was small (*N* = 26), it was not possible to make meaningful inferences about this population’s family health. Therefore, the current study only included heterosexual couples (*N* = 482). The study was approved by the university Institutional Review Board. Participants were compensated with Qualtrics credits after both members of the dyad had completed the survey.

### Measures

#### Family health

The FHS-LF [6] contains 32 items measured on a 5-point Likert scale ranging from “strongly disagree” to “strongly agree.” Negatively worded items were reversed coded so that higher scores indicated better family health. Participants were asked to report on their family based on who they considered their family to be. After answering the FHS items, participants reported whether they answered the questions based on people in their household only, people outside of their household only, or a combination of people inside and outside of their household. The family social and emotional health processes subscale contains 13 items; family healthy lifestyle includes six items; family health resources includes nine items; and family external social supports contains four items. Prior studies have demonstrated high internal reliability across all subscales, with Cronbach’s alphas for each subscale ranging from .82 to .92 [6]. The FHS-SF contains 10 items from the four subscales. Cronbach’s alpha for the current sample was .78 for women, .79 for men, and .88 for dyads.

#### Health

We examined men’s and women’s depression, executive functioning, and general health. Depression was examined using the Patient Health Questionnaire-9 (PHQ-9) [11], which uses a 4-point Likert scale (0–3 points). Internal reliability was excellent for women and men in the study (α = .94 for women; α = .93 for men). Items were summed and binary variables for men and women were created (scores > 10 were coded as moderate or severe depression). Executive functioning was measured using 15 items from the Learning, Executive, and Attention Functioning (LEAF) scale [12]. The items came from three subscales measuring attention, problem-solving, and working memory. The LEAF is on a 4-point scale (0–3 points) with higher scores indicating executive functioning deficits. Internal reliability for the LEAF measure was also excellent for both women and men in the current sample (α = .96 for women; α = .95 for men). Items were summed and binary variables measuring executive functioning deficits were created for men and women based on the clinical cutoffs proposed by the scale developers (scores of > 15 indicated a deficit) [12]. General health was measured using four items from the RAND Health Survey 1.0 [13]. Items were on a 5-point Likert scale, and negatively worded items were reverse coded so that higher scores indicated better general health. Sample items included “I seem to get sick a little easier than other people” and “I am as healthy as anybody I know.” Internal reliability was good for women (α = .73) and acceptable for men (α = .66). An average score across the four items was created for men and women.

#### Covariates

A variety of covariates were included as controls because to control for potential confounding when assessing the relationships between individual and family health status. For example, prior research indicates that childhood experiences are predictive of family health [9], and that a variety of demographic factors such as income, education, age, gender, and marital status [10] are correlated with family health. Demographic controls included women’s age in years (men’s age was not included due to the high correlation between partner age), household income, women’s and men’s race (white vs non-white), interracial marriage, and women’s and men’s education. We also controlled for childhood experiences: women’s and men’s adverse childhood experiences (ACEs) score (range 0–11) using the CDC’s BRFSS module [14]; men’s and women’s positive childhood experiences (PCEs) score (range 0–13) using the benevolent childhood experiences scale [15] and three items from the positive childhood experiences scale [16]; and women’s and men’s childhood poverty (average score from two items: “While you were growing up, about how often do you remember your family (or those you were living with) using public benefits like cash assistance, food stamps, or Medicaid?” and “While you were growing up, about how often do you remember your family receiving help from family, friends or the community to take care of basic needs?”; response options were on a 5-point scale ranging from not at all to most of the time).

### Analytic methods

Data were cleaned and item distributions examined using Stata 16. Significant differences between men’s and women’s responses were examined using the Wilcoxon signed-rank test, which is appropriate for non-parametric data. Pearson’s pairwise correlations between men’s and women’s scores across all FHS items were also examined. Confirmatory Factor Analysis (CFA) was conducted in Mplus Version 7 for both the FHS-LF and separately for the FHS-SF. For both the FHS-LF and FHS-SF, three CFA models were examined [1]. a model examining women’s and men’s responses together (unidimensional model) [2]; a correlational model where women’s and men’s responses were examined as separate factors, and [3] a second-order model where men’s and women’s responses were examined as separate factors, but a second order factor was included where the items were the factors for men’s and women’s responses. A second-order model is a higher order factor structure used to examine whether a higher order construct explains the two primary dimensions (in this case men’s versus women’s responses) [17]. A second-order model indicates that the primary dimensions of women’s and men’s responses mediates the relationship between each item and the higher order factor (family health including both dyads). For all models, error terms between men’s and women’s responses on the same items were correlated. Model fit was examined based on the root mean square error of approximation (RMSEA) and the comparative fit indices (CFI). A RMSEA < .10 indicates adequate fit, < .05 indicates good fit, and < .03 indicates excellent fit. A CFI > .90 indicates adequate fit and > .95 indicates good fit [18–20]. To determine which model was best, we examined model fit, the correlation between factor scores in the correlational model (a high correlation may indicate that the two separate factors may actually be one factor and if examined separately may increase risk for multicollinearity), and theoretical rationale.

Once the best CFA model was identified for the FHS, mean scores were calculated in Stata 16 for each subscale of the FHS-LF and the FHS-SF for men, women, and dyads. Significant differences between men’s and women’s average scores were examined using paired t-tests. Cronbach’s alphas were also computed for each subscale.

After examining different CFA models, we fit a structural model with the FHS-SF. Men’s and women’s executive functioning deficits and depression were included in the model as categorical variables. Using the Mplus “with” statement, men’s and women’s executive functioning deficits, depression, and overall health status were correlated with each other and with the FHS-SF latent variable identified during the CFA. The FHS-SF, and men’s and women’s men’s and women’s executive functioning, depression, and health were regressed on participant childhood experiences and demographic characteristics. Model fit was assessed using the same fit indices as for the CFA. All models were estimated using a robust weighted least squares estimation, which is appropriate for categorical data [21]. Full information maximum likelihood (FIML) was used to account for missing data [22] (< 1% across all items).

## Results

The sample was comprised of predominantly married couples (90.04%). About three-fourths of both women (74.90%) and men (73.44%) reported their race as white, with 12.03% of couples in an interracial relationship. Women were on average 35.64 years old and men were 38.93 years on average. Only 1.24% of women and 2.90% of men reported less than a high school education. Women and men were similar in who they considered to be their family when reporting on the FHS, with 82% of couples reporting on their family in the same way. In general, women (70.98%) and men (71.61%) reported on family members who lived in their household and did not consider family members who lived outside of their household.

Table 1 contains the mean scores for each FHS item for men and women. Mean scores did not significantly vary between men and women for any single item. The correlation between men’s and women’s scores were moderate to high (correlations ranging from .42 to .73).

**Table 1:** Item means for men and women respondents, *N* = 482

FHS Item	Mean Score (SD) Women	Mean Score (SD) Men	*P*-value for Significant Difference ^a^	Correlation Between Men and Women
1. We rarely express affection to each other. (R)	3.86(.07)	3.83(.07)	.21	.56
2. There is a feeling of togetherness.	4.41(.04)	4.46(.04)	.29	.49
3. We care for one another.	4.66(.03)	4.65(.03)	.33	.45
4. We support each other.	4.59(.03)	4.60(.03)	.39	.55
5. We rarely do things together. (R)	3.78(.07)	3.88(.06)	.65	.51
6. The things we do for each other make us feel a part of the family.	4.50(.04)	4.46(.04)	.38	.44
7. We have fun together.	4.59(.03)	4.59(.03)	.99	.46
8. We discuss problems and feel good about the solutions.	4.35(.04)	4.33(.04)	.69	.47
9. Family members pay attention to me.	4.46(.04)	4.46(.04)	.79	.41
10. Overall, I am happy with my relationship with my family members.	4.46(.04)	4.50(.04)	.12	.53
11. I feel safe in my family relationships.	4.55(.03)	4.62(.03)	.08	.44
12. We make a point of being physically active during daily life.	4.20(.05)	4.15(.05)	.37	.57
13. We usually have fresh fruits and vegetables in our home.	4.43(.04)	4.38(.04)	.11	.58
14. We help each other avoid unhealthy habits.	4.14(.04)	4.08(.05)	.26	.47
15. We make a point to follow medical recommendations.	4.36(.04)	4.31(.04)	.49	.45
16. We help each other in seeking health care services when needed (such as making doctor’s appointments).	4.41(.04)	4.35(.04)	.24	.46
17. We help each other make healthy changes.	4.35(.04)	4.32(.04)	.90	.44
18. We stay hopeful even in difficult times.	4.44(.04)	4.38(.04)	.54	.44
19. We have beliefs that give us comfort.	4.37(.04)	4.37(.04)	.64	.47
20. If we needed help from others, we would have real difficulty finding transportation to get to that help. (R)	3.14(.07)	3.18(.07)	.88	.46
21. If we needed outside help, we would not know what sort of help was available. (R)	3.28(.07)	3.40(.07)	.31	.46
22. Financial difficulties would be an obstacle to getting outside help. (R)	3.20(.07)	3.26(.07)	.25	.52
23. We do not trust doctors and other health professionals. (R)	3.98(.06)	4.04(.06)	.67	.52
24. A lack of health insurance would prevent us from asking for medical help (e.g., no health insurance or inadequate coverage). (R)	3.48(.07)	3.55(.07)	.08	.60
25. We have people outside of our family who we can turn to for help (such as for advice, help with childcare, a ride somewhere, or to borrow some money or something valuable)?	3.88(.05)	3.96(.05)	.68	.42
26. We have people outside of our family we can turn to when we have problems at school or work.	3.95(.05)	4.03(.05)	.45	.44
27. If we needed financial help, we have people outside of our family we could turn to for a loan (e.g., for $200)	3.88(.06)	3.91(.06)	.91	.60
28. If we needed help, we have people outside of our family who could provide our family with a place to live.	3.89(.06)	3.95(.06)	.50	.57
29. My MENTAL health or the MENTAL health of my family members got in the way of MY FAMILY’s normal daily activities (such as household chores, work, school, or recreation). (R)	3.27(.07)	3.38(.07)	.26	.65
30. Family worries and problems distracted me when I was working. (R)	3.48(.07)	3.59(.07)	.12	.52
31. My family did not have enough money at the end of the month after bills were paid. (R)	3.87(.07)	3.95(.06)	.08	.70
32. My family did not have adequate housing. (R)	4.36(.05)	4.38(.05)	.69	.73

^a^ Wilcoxon Signed-Rank Test. (R) = item was reverse coded

### Confirmatory factor analysis results

The unidimensional, correlational, and second-order models all had adequate and similar fit for the FHS-LF (see Table 2). The correlations between men’s and women’s factors (the correlational model) were very high for each subscale (correlations ranging from .73 to .82), indicating that this model might lead to issues with multicollinearity in a structural model. Model fit based on the RMSEA and CFI were similar between the unidimensional and second-order models, though the fit was slightly better for the second-order model. Both models would be appropriate to use in future research. Although the second-order model had slightly better fit, because fit was similar between models many researchers might choose to use the unidimensional model because it is an easier model to interpret and to create average scores for if using traditional regression software.

**Table 2:** Factor Loadings for *CFA Models for the FHS-LF for 482 Dyads*

	Unidimensional Model ^a^		Correlated Model		Second-Order Model	
	Women - λ	Men - λ	Women - λ	Men - λ	Women - λ	Men - λ
Family Social and Emotional Health Processes						
FHS 1	.54	.60	.62	.69	.64	.66
FHS 2	.74	.76	.77	.78	.77	.78
FHS 3	.76	.80	.78	.83	.78	.83
FHS 4	.81	.83	.83	.86	.83	.86
FHS 5	.50	.58	.57	.67	.57	.67
FHS 6	.79	.77	.82	.79	.82	.79
FHS 7	.71	.78	.73	.80	.73	.80
FHS 8	.73	.72	.76	.75	.76	.75
FHS 9	.76	.73	.78	.76	.78	.76
FHS 10	.77	.81	.80	.85	.80	.85
FHS 11	.81	.78	.84	.81	.84	.81
FHS 18	.72	.74	.74	.77	.74	.77
FHS 19	.66	.63	.68	.66	.68	.66
**Correlation between women and men**			.82			
**Second-Order Factor**					.92	.89
Family Healthy Lifestyle						
FHS 12	.74	.70	.77	.74	.74	.77
FHS 13	.72	.74	.74	.78	.74	.78
FHS 14	.64	.65	.66	.69	.66	.69
FHS 15	.72	.77	.75	.82	.75	.81
FHS 16	.75	.77	.77	.81	.78	.81
FHS 17	.80	.77	.82	.81	.83	.81
Correlation between women and men			.79			
Second-Order Factor					.90	.87
Family Health Resources						
FHS 20	.47	.44	.54	.51	.53	.53
FHS 21	.60	.60	.67	.66	.67	.66
FHS 22	.72	.67	.75	.69	.75	.69
FHS 23	.79	.80	.82	.83	.82	.83
FHS 24	.71	.68	.74	.70	.74	.70
FHS 29	.62	.62	.65	.65	.65	.65
FHS 30	.69	.67	.72	.69	.71	.69
FHS 31	.71	.77	.74	.79	.74	.79
FHS 32	.80	.82	.83	.85	.84	.84
Correlation between women and men			.82			
Second-Order Factor					.90	.91
Family External Social Supports						
FHS 25 (women)	.76	.81	.80	.84	.82	.82
FHS 26 (women)	.84	.86	.88	.88	.87	.89
FHS 27 (women)	.86	.89	.90	.92	.89	.92
FHS 28 (women)	.81	.78	.80	.80	.84	.81
Correlation between women and men			.73			
Second-Order Factor					.85	.86
Model Fit						
RMSEA	.056		.055		.055	
CFI	.911		.916		.914	

^a^ This is a single factor comprised of men’s and women’s scores. Items 1 and 5 and items 20 and 21 correlated error terms due to high Modification Indices and similar items. These correlated error terms could be appropriately added to all of the models

For the FHS-SF, the unidimensional model clearly had the best fit on both the RMSEA and CFI (see Table 3). As with the long-form, the correlational model for the short-form would not be appropriate as men’s and women’s factors were highly correlated (.95), indicative that the constructs are really one factor. The second-order model had adequate fit, but because the unidimensional model had much better fit it is the recommended model to use in future research.

**Table 3:** Factor Loadings for CFA Models for the FHS-SF for 482 Dyads

	Unidimensional Model ^a^		Correlated Model		Second-Order Model	
	Women - λ	Men - λ	Women - λ	Men - λ	Women - λ	Men - λ
FHS 4	.78	.75	.76	.74	.76	.74
FHS 11	.77	.77	.76	.74	.76	.74
FHS 16	.71	.72	.71	.73	.71	.73
FHS 17	.70	.66	.71	.66	.71	.65
FHS 18	.76	.72	.72	.69	.72	.69
FHS 23	.49	.54	.55	.59	.55	.59
FHS 26	.45	.47	.49	.51	.49	.51
FHS 27	.44	.49	.49	.53	.49	.53
FHS 31	.39	.46	.49	.54	.49	.54
FHS 32	.47	.47	.55	.57	.55	.57
Correlation Between Men and Women			.95			
Second-Order Factor					.96	.99
Model Fit						
RMSEA	.051		.096		.096	
CFI	.981		.927		.927	

^a^ This is a single factor comprised of men’s and women’s scores

Table 4 contains the mean scale scores for each of the FHS-LF subscales and also for the FHS-SF. Mean scores were calculated for men, women, and the total sample. Men had slightly higher scores compared to women on the family health resources subscale (*p* < .05), but there were no differences between men’s and women’s scores for the other FHS-LF subscales nor for the FHS-SF. Cronbach’s alphas ranged from .79 to .93 across all subscales, genders, and total samples (Table 4), indicating good internal reliability.

**Table 4:** Mean scores and Cronbach’s alphas for each FHS-LF subscale and the FHS-SF, by women, men, and combined scores. *N* = 482 dyads

	Mean(SD) Women	Mean(SD) Men	Mean(SD) Combined	Alpha Women	Alpha Men	Alpha Combined
FHS – Short Form	4.24(0.62)	4.26(0.63)	4.25(0.58)	.79	.80	.88
Family Social and Emotional Health Processes	4.39(0.59)	4.39(0.61)	4.39(0.56)	.87	.89	.93
Family Healthy Lifestyle	4.31(0.67)	4.27(0.73)	4.29(0.64)	.82	.85	.90
Family Health Resources	3.56(1.00)	3.64(0.96)	3.60(0.92)*	.85	.86	.92
Family External Social Supports	3.90(1.03)	3.96(1.00)	3.93(0.92)	.88	.88	.90

* Significant difference in scores between men and women, *p* < .05

### Structural model of associations between participant characteristics and family health

The structural model (RMSEA: 0.039; CFI: 0.947) provided some information on what individual characteristics are most associated with family health (using the unidimensional FHS-SF latent variable). Men’s and women’s executive functioning deficits and depression were negatively correlated with family health. Higher women’s and men’s health scores were correlated with better family health. Higher household income and women’s and men’s PCEs were all associated with better family health. More ACEs for men were associated with lower family health; women’s ACEs were not associated with later family health. No other demographic variables were associated with family health. Table 5 contains the full results of the structural model.

**Table 5:** Structural Equation Model of the Relationships Between Individual and Family Health. N = 482

	Family Health	Women’s EF Deficits	Men’s EF Deficits	Women’s Depression	Men’s Depression	Women’s General Health	Men’s General Health
**Adjusted Correlations**							
Women’s EF Deficits	−.57***	–					
Men’s EF Deficits	−.44***	.53***	–				
Women’s Depression	−.55***	.69***	.49***	–			
Men’s Depression	−.40***	.53***	.49***	.61***	–		
Women’s Health	.32***	−.40***	−.27***	−.33***	−.27***	–	
Men’s Health	.33***	−.31***	−.40***	−.21***	−.43***	.26***	–
**Structural Equation Model Controls**							
Women’s Age	−.00	−.06	.01	.09	.05	−.12**	−.12**
Household Income	.23***	−.04	−.04	−.16*	−.10	.19**	.15*
Women’s Race (White)	−.05	.04	–	−.06	–	−.18**	–
Men’s Race (White)	.00	–	−.05	–	−.02	–	−.08
Interracial Marriage	−.02	−.02	−.04	−.03	−.00	−.01	−.00
Women’s Education	−.03	−.22*	–	.02	–	−.04	–
Men’s Education	.04	–	.07	–	.21	–	−.11
Women’s ACEs	−.06	.32***	–	.42***	–	−.25***	–
Men’s ACEs	−.22***	–	.28	–	.43***	–	−.29***
Women’s PCEs	.19***	−.09	–	−.06	–	.12**	–
Men’s PCEs	.22***	–	−.11	–	−.13	–	.11*
Women’s Childhood Poverty	−.10	.02	–	.04	–	.00	–
Men’s Childhood Poverty	−.02	–	.04	–	.09	–	−0.07

*p < .05. **p < .01. ***p < .001. Model Fit: RMSEA: 0.039; CFI: 0.947. Notes: EF = executive functioning deficits; EF was measured using the LEAF scale; depression was measured using the PHQ-9; men’s and women’s health was measured using the RAND Health Survey; and family health was measured using the FHS-SF

## Discussion

The results demonstrate that the FHS is a valid and reliable family measure when examining family health among married and cohabitating heterosexual couples who have children. The unidimensional factor structure was the strongest factor structure for the FHS-SF while either the unidimensional model or the second-order model would be appropriate for the FHS-LF. Correlational factor structures would not be appropriate to use for either the FHS-LF or FHS-SF due to high correlations between men’s and women’s factor scores. The results suggest that men and women report on their family health similarly.

Results from the structural model indicated that individual health was correlated with the family’s health as a whole in the expected direction. Prior research has also shown a strong correlation between depression and executive functioning with family health [6, 10]. The correlation coefficients in the current model appear larger than prior studies. This is in part because we modeled depression and executive functioning as binary variables in the current study (e.g., presence of depression or executive functioning deficits) rather than as continuous variables. Individual general health was also correlated with family health, but the correlation coefficients were smaller, indicating a slightly weaker association between the family’s health and individual perception of their overall health. Based on the items in the general health measure, participants may have considered their physical health more than their mental health when answering the general health items. Thus, family health may be more strongly associated with mental health than with physical health, a concept that should be explored further in future studies. Because the data were cross-sectional, it is not possible to definitively determine whether family health affects individual health, individual health affects family health, or whether the relationship between individual and family health is bidirectional. Longitudinal data collection is an essential next step to uncovering what helps make families healthy.

Consistent with the hypothesis, most demographic controls were not strongly associated with family health, suggesting that families are healthy across a variety of contexts. Higher household income was modestly associated with better family health, likely because it increases resources and decreases other stressors. PCEs were linked with better family health in adulthood, which is consistent with prior studies using the long-form that indicated that regardless of ACEs, positive childhood experiences led to better family health in adulthood [9].

There were important gender differences in how individual characteristics affected family health. First, the standardized correlation coefficients for women’s mental health (as measured by depression and executive functioning) with family health were slightly larger compared to men’s mental health with family health. Although this fits the adage that “when mamma ain’t happy, ain’t nobody happy,” the nuances of this deserve more attention. Prior research has indicated that the combined depression for both mothers and fathers leads to increased emotional and behavioral problems in children; this effect was still realized when mother’s alone were depressed but not when father’s alone were depressed [23]. The current study showed a correlation with family health for both mothers and fathers depression, suggesting that changing gender roles in family life may implicate father’s health in family wellbeing more so than in the past [24], though women’s mental health may still be disproportionately important. Second, men’s ACEs (but now women’s ACEs) were predictive of worse family health. The reasons for this are not entirely clear. Prior research has demonstrated that fathers with higher ACEs were more likely to experience more conflict with the mother of their child compared to fathers with lower ACEs [25]. However, other research has found that both men’s and women’s childhood trauma have similar effects on relationship quality in adulthood [26]. Further longitudinal research examining the respective influence of father’s ACEs and mother’s ACEs on different dimensions of family health (e.g., relationships, material resources, coping, familial lifestyle behaviors, etc.) is an important next step.

Limitations of the current study include that the sample was not representative of all couples in the United States. In particular, same-sex couples were not represented in the current study, and heterosexual couples with low education were underrepresented. Future studies using the scale among same-sex couples and couples with low education is important. Additionally, data were cross-sectional and causality between male and female characteristics compared with family health should not be inferred. Finally, all results are based on participant self-report. This is considered gold standard for some indicators such as when measuring depression, but task-based or observed indicators are typically more accurate for measuring executive functioning and other health variables.

Despite these limitations, the results of the study do provide information on the reliability and validity of the FHS among couples dyads and provide some context for the correlation between individual characteristics with family health. Future studies should include longitudinal data to better understand trends in family health over time and adolescent participants in an effort to validate the FHS among youth, as currently the FHS has only been validated among adults.

## Data Availability

The datasets generated and/or analyzed during the current study are not publicly available due to IRB requirements but are available from the corresponding author on reasonable request.

## Abbreviations

ACE: Adverse Childhood Experience
CFA: Confirmatory Factor Analysis
CLI: Comparative Fit Indices
FHS: Family Health Scale
FHS-LF: Family Health Scale – Long Form Version
FHS-SF: Family Health Scale – Short Form Version
FIML: Full Information Maximum Likelihood
LEAF: Learning, Executive, and Attention Functioning
PHQ: Patient Health Questionnaire
PCE: Positive Childhood Experience
RMSEA: Root Mean Square Error of Approximation
